# The Positive Association between Melatonin-Containing Food Consumption and Older Adult Life Satisfaction, Psychoemotional State, and Cognitive Function

**DOI:** 10.3390/nu16071064

**Published:** 2024-04-05

**Authors:** Mikhail F. Borisenkov, Olga I. Dorogina, Sergey V. Popov, Vasily V. Smirnov, Anna A. Pecherkina, Elvira E. Symaniuk

**Affiliations:** 1 Department of Molecular Immunology and Biotechnology, Institute of Physiology of Federal Research Centre Komi Science Centre, Urals Branch of the Russian Academy of Sciences, 50 Pervomaiskaya Str., 167982 Syktyvkar, Russia; s.v.popov@inbox.ru (S.V.P.); smirnowich@yandex.ru (V.V.S.); 2 Ural Institute of Humanities, Ural Federal University, 51 Lenina Str., 620000 Yekaterinburg, Russia; 79apa@mail.ru (A.A.P.); e.e.symaniuk@urfu.ru (E.E.S.)

**Keywords:** food melatonin, life satisfaction, depression, cognitive function, older adults

## Abstract

The purpose of this study was to test the hypothesis that melatonin-containing food (FMT) consumption is associated with a better sleep schedule and cognitive and psychoemotional state in older adults. A cross-sectional study of 557 (79% females) older adults living in the community with a mean age of 68.9 ± 7.7, ranging from 50 to 90 years, was conducted. The study, conducted in May and September 2023 using a face-to-face interview, collected personal data and assessed FMT intake during the day (FMT_day_) and for dinner (FMT_dinner_), life satisfaction, positive and negative affect, depression severity, cognitive functions, and sleep characteristics. Multiple regression and logistic regression analysis, adjusted for co-factors, were used to assess the association between the studied indicators. Multiple regression analysis showed that older adults with higher FMT consumption are more satisfied with life (FMT_dinner_: *β* = 0.107; ∆*R*^2^ = 0.011; *p* = 0.020), have a lower level of depression (FMT_day_: *β* = −0.124; ∆*R*^2^ = 0.015; *p* = 0.003), and higher scores in positive affect (FMT_day_: *β* = 0.169; ∆*R*^2^ = 0.016; *p* = 0.007; FMT_dinner_: *β* = 0.136; ∆*R*^2^ = 0.019; *p* = 0.003). Logistic regression analysis showed that older adults with higher FMT consumption are less likely to have depression (FMT_day_: OR, 0.614; 95% CI, 0.436–0.864; *p* = 0.005; FMT_dinner_: OR, 0.671; 95% CI, 0.476–0.945; *p* = 0.023), and they perform better on logical thinking tests (FMT_day_: OR, 2.066; 95% CI, 1.131–2.204; *p* = 0.013; FMT_dinner_: OR, 1.887; 95% CI, 1.183–2.138; *p* = 0.033). A greater life satisfaction as well as a decrease in the cognitive impairment and psychoemotional state of older adults is associated with a higher consumption of melatonin-containing foods.

## 1. Introduction

Due to the steady increase in life expectancy and the associated increase in the share of older adults in modern society, the task of maintaining their health is becoming more urgent, which is a required condition for improving the quality of life and life satisfaction of older adults [1,2]. To a large extent, the life satisfaction of older adults also depends on their psychoemotional state [3,4,5], the degree of preservation of cognitive functions [5,6], and sleep quality and quantity [3,4]. 

Along with a significant improvement in the quality of human life by reducing the negative impact of adverse climatic conditions and the development of medicine, a number of new factors have appeared in modern society that disrupt the function of the circadian system (CS) and, thus, have a negative impact on the psychophysiological state and human health throughout a person’s life. The use of artificial lighting at night [7] resulted in a weakening of the synchronization of the circadian clock with the 24 h rhythms of the environment. The introduction of time zones all over the planet [8], and in some countries, an increase in their size [9], as well as the introduction of annual daylight-saving time in many countries [10] has led to an increased risk of jetlag caused by a mismatch between solar, social, and biological clocks [11]. Large groups of people have to work in rotating or night shift schedule for many years, which results in a mismatch between social and biological rhythms [12]. Socially active people regularly make transmeridian flights, being exposed to jetlag [13]. Starting work/school too early is the reason for the development of social jetlag, a form of CS misalignment, most often observed in people with a late chronotype [11,14]. All these and many other factors lead to chronic dysfunction of the circadian system, accompanied by the deterioration of the psychoemotional state [15], cognitive functions [16], and an increased risk of obesity [17], cardiovascular [18], neurodegenerative [19], and oncological diseases [20,21]. Thus, CS dysfunction is one of the reasons for a decrease in the quality of human life in modern society. Therefore, an urgent task is to find and put into practice methods and tools that ensure the preservation of the human CS function throughout a person’s life [22].

Some progress is made in using the principles of chronotherapy in the treatment of certain diseases, such as depression [23] and oncological diseases [24]. Tools for the prevention of CS function disorders are being developed. One of the effective means of preventing the CS function disorders is the application of the principles of chrononutrition [25]. In particular, there is a close association between the stability of the daily rhythm of eating and the psychoemotional state of young people [26]. The use of food products containing chronobiotics, a class of substances that affect the function of the CS [27], to stabilize circadian rhythms has also shown its effectiveness. 

An inverse relationship between the consumption of melatonin-containing foods (FMT) and mortality from all causes in adults was found [28]. Schoolchildren and students with higher consumption of FMT are less likely to experience social jetlag, sleep disorders, and the deterioration of their psychoemotional state [29]. However, it should be noted that these results are mainly obtained in young adults. An urgent task is to clarify the question of how effective the application of the principles of chrononutrition is to improve the psychophysiological state of the older population. The purpose of this study was to test the hypothesis that the consumption of FMT is positively associated with life satisfaction, psychoemotional state, sleep schedule, and the cognitive function of older people.

## 2. Methods

### 2.1. Study Population and Data Collection

Older adults living in the community (i.e., not living in nursing homes or assisted living facilities) voluntarily participated in the study conducted in May and September 2023. Ural Federal University psychology students interviewed their relatives (~90%), as well as older adults in socio-cultural associations and pensioners’ associations (~10%). The data were entered into spreadsheets. It should be noted that in our study, all subjects were volunteers with a certain level of involvement, continued activity, interest, and motivation, which puts them at an average socio-economic level. All participants signed informed consent and could withdraw from participation in the study at any time. A total of 557 people took part in face-to-face interviews; the average age was 68.9 ± 7.7, ranging between 51 and 90 years, with 79% being female. More than 80% of the study participants live in Yekaterinburg (lat.: 56.9°; long.: 36.6°; population: 1,469,000), Sverdlovsk, and the regions closest to it. 

Each study participant indicated their personal data (place of residence, education, profession, work experience, sex, age, height, weight, and waist circumference) and completed a series of tests to assess their psychophysiological condition. The psychoemotional state was assessed using the Satisfaction with Life Scale (SWLS) [30], the Positive and Negative Affect Schedule (PANAS) [31], and the Zung Self-Rating Depression Scale (ZSDS) [32]. Cognitive functions were assessed using the Rapid Cognitive Screen (RCS) test [33]. Sleep characteristics and quality were assessed using the Munich Chronotype Questionnaire (MCTQ) [34] and the Pittsburgh Sleep Quality Index (PSQI) [35]. In addition, all study participants completed a modified food frequency questionnaire (FFQ) to estimate the frequency of food intake, which assessed the consumption of FMT, as described earlier [29]. The height, weight, and waist circumference indicated by the study participants were used to calculate BMI and the waist-to-height ratio (WHtR).

This study was approved by the Ethics Committee of the Institute of Physiology of the Komi Science Centre of the Ural Branch of the Russian Academy of Sciences (21 September 2020). Verbal informed consent was obtained from all study participants. 

### 2.2. Instruments

#### 2.2.1. The Satisfaction with Life Scale (SWLS)

To estimate life satisfaction, an adapted SWLS scale was used and translated into Russian [36]. SWLS [30] consists of 5 statements regarding life satisfaction (for example, “basically my life is close to ideal”). An interviewee is asked to assess the degree of agreement with these statements by giving the appropriate score from 1 (I completely disagree) to 7 (I completely agree). The sum of the points, ranging from 5 to 35, is used as an assessment of the degree of life satisfaction. In our sample, the mean values and standard deviations M (SD) for the SWLS score was 24.7 (5.9). Cronbach’s α for this sample was 0.804.

#### 2.2.2. The Positive and Negative Affect Schedule (PANAS)

An adapted and shortened version of PANAS in Russian [37] was used. PANAS [31] is a scale widely used to estimate mood or emotions. The abbreviated Russian version of PANAS [37] consists of two sub-scales, each of which contains 6 statements for evaluating positive (e.g., excitement or inspiration) and negative affect (e.g., frustration or fear), respectively. Each item is rated on a 5-point scale from 1 (not at all) to 5 (very strongly). The mean values and standard deviations M (SD) for positive and negative affect in our sample were 17.6 (4.4) and 12.0 (5.1) respectively. Cronbach’s α for the positive affect sub-scale was 0.787, and for the negative affect sub-scale, 0.862.

#### 2.2.3. The Zung Self-Rating Depression Scale (ZSDS)

The level of depression was assessed by self-assessment using the ZSDS test [32]. The ZSDS is a 20-item self-report measure of depressive symptom severity. The answer options (scores) are as follows: none or little of the time (1), some of the time (2), good part of the time (3), and most of the time (4). When processing questionnaires, the total of the raw scores is calculated, varying in the range from 20 to 80. The raw scores are then recalculated into ZSDS indices (ZSDSIs) according to the methodology described in Zung [38] and Passik et al. [39]. The ZSDSI is a continuous variable ranging from 25 to 100, representing a quantitative assessment of the depression severity. The average values and standard deviations M (SD) for ZSDSI in our sample were 44.9 (11.1). This indicator was used in covariance and multiple regression analyses. Qualitative assessment of the depression severity was obtained by dividing these data into four categories: I—no depression (ZSDSI ≤ 50); II—minimal to mild depression (ZSDSI 51–59); III—moderate to significant depression (ZSDSI 60–69); and IV—severe to extreme depression (ZSDSI ≥ 70). In the logistic regression analysis, two levels of depression (no/yes) were distinguished, while the frequencies were summarized for two pairs of categories (I + II/III + IV). Cronbach’s α for this sample was 0.848.

#### 2.2.4. The Rapid Cognitive Screen (RCS)

The RCS [33] presents a rapid cognitive screen test that is used to assess cognitive abilities in older people in various settings. It is most often used for the rapid screening of dementia in older adults caused by Alzheimer’s disease or other neurodegenerative diseases. RCS consists of a short-term memory test (RCS Memory), which consists of the ability to reproduce five words; a clock drawing test (RCS Clock); and a logic thinking test (RCS Logic), consisting of the ability to remember a story and convert the fact that Kyiv is in Ukraine [33]. The adapted Russian translation of RCS [40] was used in the study. Cronbach’s alpha value of the RCS was 0.713.

#### 2.2.5. The Munich Chronotype Questionnaire (MCTQ)

The MCTQ [34] was used to estimate the sleep–wake rhythm characteristics. Interviewees are proposed to describe the usual sleep–wake schedule during the month preceding the study, on weekdays and weekends separately. The interviewees are asked to indicate the time when they go to bed, when they finally fall asleep, how long it takes them to finally fall asleep, when they wake up, when they get out of bed, how long it takes them to finally wake up, and whether they use an alarm clock. Using these data, the average weekly sleep duration, sleep efficiency, chronotype, and social jetlag were calculated using formulas described in detail previously [41].

#### 2.2.6. The Pittsburgh Sleep Quality Index (PSQI)

The Russian version of PSQI [42] was used to estimate the quality of sleep. The test contains items related to the assessment of sleep characteristics over a month preceding the survey, the time to fall asleep, inertia, duration, sleep efficiency, as well as a self-report of sleep quality, problems with falling asleep, daytime sleepiness, the use of sleeping pills, etc. The total sum of PSQI scores is a continuous variable, with the mean and standard deviation M (SD) equal to 6.65 (2.90) in our sample, ranging between 0 and 15 in scores. We used this variable as a quantitative assessment of sleep quality. In the logistic regression analysis, a qualitative assessment of the indicator was used, which, according to the recommendation of the authors [35], has two categories: 1. PSQI score of ≤5 corresponding to good sleep quality, and 2. PSQI score of >5 corresponding to poor sleep quality.

#### 2.2.7. FMT Consumption

To assess FMT consumption, a modified test for estimating the frequency of food consumption (FFQ) was used. 

The only MT-containing foods (according to the literature) were included in the questionnaire. The list of foods included in this questionnaire is available in the Supplementary Materials to the paper [29]. The study participants were asked to answer three questions as follows:

1. How often have you consumed these foods in the past month? Answer options [conversion factor for estimating frequency of consumption per day]: never (0), 1–2 times a month (0.05), 3–4 times a month (0.12), 2–3 times a week (0.36), 4–6 times a week (0.71), 1–2 times a day (1.5), 3–4 times a day (3.5), and more than 4 times a day (5).
2. How many servings of these foods did you consume in one meal (this question was accompanied by a picture indicating the size of one serving and the product’s weight in grams)? Answer options: 0.5, 1, 2, 3, 4, or 5 servings.
3. What percentage of the foods above was eaten during dinner? The response options were 0, 25, 50, 75, or 100%.

These data were used to calculate FMT consumption per day (FMT_day_, ng/day) and per dinner (FMT_dinner_, ng/dinner) by multiplying the average number of FMT consumed per day and at dinner by the average MT content (ng/g) in those products. A detailed methodology for calculating indicators is available in the Supplementary Materials to the paper [29]. 

### 2.3. Statistical Analysis

The SPSS 20 software package (SPSS, Inc., Chicago, IL, USA) was used in analyses. The mean, standard deviation, asymmetry, and kurtosis were found for quantitative variables, and percentages were found for qualitative ones. Covariance and multiple regression analyses, adjusted for related factors, were used to analyze the association between quantitative indicators. Indicators obtained using SWLS, PANAS, ZSDS, RCS, MCTQ, and PSQI tests were used as dependent variables, and FMT_day_ and FMT_dinner_ adjusted for sex, age, BMI, season, education level, and social status were used as independent variables. Preliminary analysis showed that the distribution of FMT_day_ and FMT_dinner_ samples differ from normal; therefore, categorical values based on tertiles were used for the analysis (see details in Supplementary Information Table S1). To assess the association between qualitative indicators, the criterion χ^2^ and logistic regression analysis were used. When conducting logistic regression analysis, indicators obtained using ZSDS, RCS Logic, MCTQ, and PSQI, expressed in categorical form, were used as dependent variables. The same indicators were used as independent variables and covariates as in the multiple regression analysis.

## 3. Results

The average age of the study participants was, represented as M (SD), 68.9 (7.7) years. The average values of anthropometric indicators were 27.95 (5.24) for BMI and 0.56 (0.11) for WHtR. The average score on the life satisfaction scale was 24.7 (5.9) points, and positive and negative affect scores were 17.6 (4.4) and 12.0 (5.1), respectively. The average value of social jetlag in the study participants was 0.27 (0.78) hours, sleep duration was 7.5 (1.4) hours, and sleep efficiency was 87.7 (8.7)%. The study participants consumed, on average, 1916.2 (2548.16) ng/day for FMT_day_ and 675.2 (1527.26) ng/dinner for FMT_dinner_.

A total of 79% of the study participants were women (Table 1). A proportion of 52.6% had secondary and specialized secondary education, and 34.5% had higher and postgraduate education. A total of 62% of the study participants were retired, and 38% worked. A total of 68.5% of the study participants were overweight or obese, and 69% had signs of visceral obesity. A social jetlag of more than 1 h was noted by 18% of respondents. A total of 51.5% of respondents had a normal sleep duration (7–8 h), while 19% and 29.5% had insufficient (less than 6 h) and excessive (more than 9 h) sleep duration, respectively. Poor sleep quality was noted in 54.6% of the people we interviewed. Signs of depression were noted in 11.5% of respondents. There are significant sex differences in education, occupation, BMI, social jetlag, sleep duration, sleep quality, and the level of depression (Table 1).

**Figure 1:**
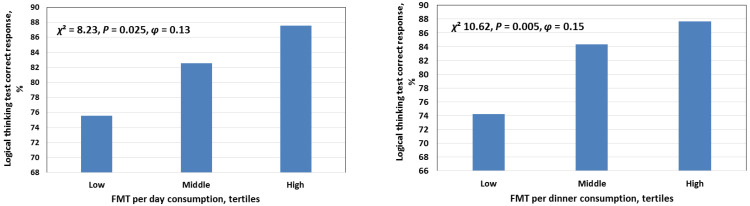
Association between melatonin-containing food consumption for day (**left panel**) and for dinner (**right panel**) and frequency of logical thinking test correct response in older adults. *χ*^2^—chi-squared test; *φ*—Cramer’s V effect size.

According to the analysis of covariance, individuals with higher consumption of FMT have a higher score on the SWLS scale (FMT_day_: *F* = 4.67; *p* = 0.010; partial *η*^2^ = 0.019; FMT_dinner_: *F* = 4.16; *p* = 0.016; partial *η*^2^ = 0.018) and the PANAS sub-scale of positive affect (FMT_day_: *F* = 3.97; *p* = 0.019; partial *η*^2^ = 0.016; FMT_dinner_: *F* = 5.85; *p* = 0.003; partial *η*^2^ = 0.025). There was also an inverse relation between FMT consumption and the level of depression (FMT_day_: *F* = 7.56; *p* = 0.006; partial *η*^2^ = 0.014; FMT_dinner_: *F* = 5.86; *p* = 0.016; partial *η*^2^ = 0.011). No association between FMT consumption and anthropometric indicators of older adults (*p* > 0.05) were found. Older adults with a higher consumption of FMT have higher sleep quality (FMT_day_: *F* = 4.10; *p* = 0.043; partial *η*^2^ = 0.008) and have longer sleep duration (FMT_dinner_: *F* = 2.68; *p* = 0.021; *η*^2^ = 0.025). Older individuals with a higher consumption of FMT have higher memory test scores (FMT_day_: *F* = 3.34; *p* = 0.010; partial *η*^2^ = 0.027; FMT_dinner_: *F* = 4.48; *p* = 0.001; partial *η*^2^ = 0.038) and logical thinking (FMT_day_: *χ*^2^ = 8.23; *p* = 0.025; *φ* = 0.129; FMT_dinner_: *χ*^2^ = 10.62; *p* = 0.005; *φ* = 0.150) (Figure 1).

Using multiple regression analysis, it was shown that older adults with higher FMT intake are more satisfied with life (FMT_dinner_: *β* = 0.107; ∆*R*^2^ = 0.011; *p* = 0.020), have a lower level of depression (FMT_day_: *β* = −0.124; ∆*R*^2^ = 0.015; *p* = 0.003), and more high scores on the positive affect scale (FMT_day_: *β* = 0.169; ∆*R*^2^ = 0.016; *p* = 0.007; FMT_dinner_: *β* = 0.136; ∆*R*^2^ = 0.019; *p* = 0.003; Table 2).

Using logistic regression analysis, it was found that older adults with a higher FMT intake have a lower level of depression (FMT_day_: OR, 0.614; 95% CI, 0.436–0.864; *p* = 0.005; FMT_dinner_: OR, 0.671; 95% CI, 0.476–0.945; *p* = 0.023) and higher results in the assessment of logical thinking (FMT_day_: OR, 2.066; 95% CI, 1.131–2.204; *p* = 0.013; FMT_dinner_: OR, 1.887; 95% CI, 1.183–2.138; *p* = 0.033; Table 3). 

## 4. Discussion

For the first time, we noted a positive association of FMT consumption with life satisfaction, positive affect, and logical thinking in older adults. Moreover, the results of logical thinking testing are positively associated with FMT_day_ and FMT_dinner_, adjusted for related factors, including the level of education. Life satisfaction is an integral indicator reflecting the social and psychophysiological state of a person [1,2,3,4,5,6]. The association we noted between FMT_dinner_ and SWLS suggests that melatonin-containing food consumption has a general stimulating effect on all functions of the human body. Previously, some authors [43] have shown that melatonin is a geroprotector. However, not all studies note the geroprotective properties of melatonin [44]. Nevertheless, it is shown that MT reduces the risk of developing oncological [43], cardiovascular [45], and neurodegenerative [46] diseases and, as a result, increases the duration of a healthy life [44]. Until recently, a pharmacologically pure melatonin preparation was used in gerontological studies. However, a recent study has shown for the first time that FMT consumption also has a positive effect on human health, reducing the risk of death from all causes [28]. The data presented in our study suggest that the main targets of the action of dietary melatonin are the brain regions responsible for the psychoemotional state and cognitive functions of older adults. In the future, it is necessary to study, in more detail, the health protective properties of dietary melatonin, the undoubted advantage, of which in comparison with a pharmacologically pure drug, is its cheapness, ease of use, and the absence of any special restrictions in use.

The noted neuroprotective properties of dietary melatonin are most likely related to the repeatedly described ability of melatonin to prevent the accumulation of amyloid in the hippocampus and, thus, slow down the age-associated decline in human cognitive functions [47]. It is shown that the neuroprotective effect of melatonin in transgenic mice predisposed to the formation of amyloid plaques was observed with chronic administration of melatonin, starting with 4 months of age [48]. If melatonin was administered to transgenic mice predisposed to Alzheimer’s disease starting at the age of 14 months, the neuroprotective effect of the drug was not observed [49]. Alzheimer’s disease is a chronic disease that has been developing for many years. In adults, after the age of 50, long before the clinical manifestation of Alzheimer’s disease, cognitive impairment is detected while maintaining daily activities, and is diagnosed as mild cognitive impairment (MCI) [50]. In studies [50,51], it was shown that patients with MCI with prolonged (from 15 months to 5 years) oral administration of melatonin (3–24 mg) showed an improvement in cognitive functions. The food preferences of adults and older adults are quite stable, especially in relation to fruits and vegetables [52]. It can be assumed that the participants in our study also consumed FMT for many years, and, thus, were chronically exposed to dietary melatonin, which slows down age-associated cognitive function decline.

The positive association between FMT consumption and psychoemotional state noted in this study, as well as in our previous study with the participation of young adults [29], is most likely due to the fact that melatonin belongs to chronobiotics [27]. It is known that exogenous melatonin is able to change the phase of the endogenous circadian rhythm and, thus, have a therapeutic effect on patients with various forms of depression [53]. A comparative analysis of the incidence of depression assessed using the same tool (ZSDS) in young adults [29] and in older adults (Table 1) showed that (a) in young adults, the incidence of moderate/severe depression is higher than in older adults (18.1 vs. 11.5%), and (b) the effect size (∆*R*^2^) of the association between FMT_day_ and ZSDSI in young adults was lower than in older adults (0.012 vs. 0.015). In general, these data indicate that older adults have less symptoms of depression and that their psychoemotional state is more associated with the consumption of FMT. Previously, it has been repeatedly noted that the frequency of detecting symptoms of depression decreases with age [54]. Moreover, it was shown [54] that age-associated changes in the frequency of depression detection are most pronounced in women. Since women predominated in our two studies (72% and 79%, respectively), this explains the presence of pronounced age-related dynamics in the frequency of depression detection. In future studies, it is necessary to study, in more detail, the assumption that dietary melatonin is potentially a more effective means of preventing depression in female older adults.

No association between dietary melatonin intake and sleep quality in older adults was found. No association between diet and sleep duration and the frequency of detection of social jetlag was found as well. The associations were found in young adults [29] in our previous study. Differences in the results of the study seem to be related to differences in the age of the compared groups. In the older adults interviewed in this study, social jetlag was noted only in 18% of respondents, and the average values of the indicator were 0.27 (0.78) hours, whereas in young adults, similar indicators were 53.2% and 1.21 (1.26) hours, respectively [29]. The lower values of the social jetlag in older adults are explained by the fact that, as a rule, at an older age, there is less delay in the sleep–wake rhythm phase, and they are less involved in performing social duties. It is known that the late chronotype and the too early start of classes/work are the two main (but not the only) factors, the combination of which leads to an increased risk of developing social jetlag [11]. Moreover, it has been repeatedly shown that social jetlag is more often detected in adolescents and young adults [11,14]. It should be noted that in this study, we have considered only one of the possible forms of circadian misalignment. Social jetlag is caused by a mismatch between social, solar clocks, and the sleep–wake rhythm [11]. In older adults, one of the main reasons for the circadian misalignment is the central clock dysfunction. They have a general decrease in melatonin production by the epiphysis [55] and a decrease in the amplitude of the circadian rhythm of the hormone in the blood [56], resulting in a decrease in the ability of the central clock to synchronize circadian rhythms in peripheral organs [57].

The consumption of energy with food decreases with age [58], so we may assume that the consumption of melatonin with food may also decrease with age. A comparative analysis of the food melatonin content in the diet of young adults [29] and older adults showed that, indeed, young adults consume 13.3% more FMT_day_ and 24.8% more FMT_dinner_ than older adults. In further studies, it is necessary to study the association between the consumption of melatonin-containing food with disorders of the psychophysiological parameters of older adults due to internal desynchrony. 

No association between dietary melatonin intake and anthropometric indicators (BMI and WHtR) of older adults was found, whereas in young adults who consume more FMT, a decrease in the incidence of visceral obesity was found [29]. Comparative analysis showed that in young adults [29], the detection rate of visceral obesity is about six times lower (11.1% vs. 69.1%) than in older adults (Table 1). Circadian misalignment, which reaches its maximum at the age of 20 [11], appears to significantly contribute to an increased risk of obesity in young adults [17]. Therefore, dietary melatonin, acting as an additional external synchronizing signal for the circadian system, has a beneficial effect on their risk of developing obesity. In older adults, the main cause of obesity is a decrease in the level of motor activity [59], which explains the lack of association between dietary melatonin intake and anthropometric indicators.

The conducted research has a number of advantages and limitations. The advantages of the study include the complex, interdisciplinary nature of the study, in which a number of anthropometric, physiological, and psychological indicators were evaluated in the same group of older adults. An important advantage of the study is the fact that the studied indicators were evaluated when the study participants were in natural conditions. Psychology students conducted a face-to-face interview of their grandparents, which significantly reduced the risk of some errors caused by interviewer effects [60]. Limitations include the fact that the psychoemotional state, life satisfaction, and the sleep schedule were measured by self-assessment, which significantly reduces the accuracy of the assessments of the study participants’ condition. The consumption of FMT was assessed based on the respondents’ retrospective memories of their diet over the past month, which could result in a decrease in the accuracy of estimates in cases where the study participants had memory problems. The cross-sectional design used in this study does not allow us to judge the causal relationship between the studied indicators.

## 5. Conclusions

The results of this cross-sectional study confirmed our assumption that older adults with higher FMT consumption are more satisfied with life, less likely to notice a deterioration in their psychoemotional state, and cope more easily with tests for cognitive functions. Thus, the presented results suggest that the chrononutrition principles may be used to improve the psychophysiological state of the older population.

## Figures and Tables

**Table 1:** Categorical variables and sex differences.

Variables	Gradations	*N*	%	F, %	M, %	*χ* ^2^	*p*	*φ*
Sex	F	440	79	100	-			
	M	117	21	-	100			
Education	Lower secondary	15	2.69	2.73	2.56			
	Compete secondary	57	10.23	11.36	5.98			
	Primary special	279	50.09	49.09	53.85			
	Secondary special	14	2.51	2.27	3.42			
	Higher	176	31.60	32.73	27.35			
	Postgraduate	16	2.87	1.82	6.84	12.48	0.05	0.15
Occupation	Retired	342	61.73	65.67	47.00			
	Retired + work	110	19.86	18.54	24.79			
	Work	102	18.41	15.79	28.21	14.75	0.001	0.16
BMI categories	Underweight	7	1.26	0.68	3.45			
	Normal weight	168	30.22	30.00	31.03			
	Overweight	199	35.79	38.41	25.86			
	Obesity	182	32.73	30.91	39.66	11.80	0.01	0.15
Visceral obesity (WHtR ≥ 0.5)	0	167	30.93	31.07	30.36			
	1	373	69.07	68.93	69.64	0.02	n.s.	0.01
Social jetlag (SJL ≥ 1 h)	0	457	82.04	78.18	64.96			
	1	100	17.96	21.82	35.04	8.72	0.005	0.13
Sleep duration categories	≤6 h	105	18.85	20.91	11.11			
	7–8 h	287	51.53	50.91	53.85			
	≥9 h	165	29.62	28.18	35.04	6.33	0.05	0.11
PSQIc categories	0 (good)	254	45.36	41.82	58.97			
	1 (bad)	306	54.64	58.18	41.03	10.97	0.001	0.14
ZSDSIc categories	0	493	88.51	86.82	94.87			
	1	64	11.49	13.18	5.13	5.89	0.025	0.10
RCS (logical thinking test)	0	89	18.02	18.69	15.31			
categories	1	405	81.98	81.31	84.69	0.61	n.s.	0.04

A chi-squared test was used to analyze sex difference between variables; F—females; M—males; *χ*^2^—chi-squared test; *φ*—Cramer’s V effect size; n.s. —not significant.

**Table 2:** Results of multiple regression analyses.

#	Dependent Variable	Predictors	*B*	*β*	*R* ^2^	∆*R*^2^	*p*	*VIF*
1	SWLS	FMT_dinner_	0.762	0.107	0.011	0.011	0.020	1.000
2	ZSDSI	Age	0.016	0.164	0.029	0.029	0.000	1.015
		Sex	0.204	0.111	0.041	0.012	0.008	1.013
		FMT_day_	−0.113	−0.124	0.056	0.015	0.003	1.002
3	PA	Age	−0.081	0.026	0.016	0.016	0.002	1.013
		FMT_day_	0.458	0.169	0.031	0.015	0.007	1.013
4	PA	Age	−0.078	−0.136	0.019	0.019	0.003	1.000
		FMT_dinner_	0.386	0.136	0.038	0.019	0.003	1.000

A series of multiple regression analyses were performed in which life satisfaction (SWLS), positive affect (PA), depression (ZSDSI), while melatonin-containing food consumption per day (FMT_day_) in tertiles (codes: 1—low, 2—middle, 3—high; see details in Table S1), melatonin-containing food consumption per dinner (FMT_dinner_) in tertiles (codes: 1—low, 2—middle, 3—high; see details in Table S1), age, sex (codes: 1—males, 2—females), males—reference group, BMIc weight categories (codes: 1—underweight, 2—normal weight, 3—overweight, 4—obese), season (codes: 1—spring, 2—autumn), education categories (codes: 1—lower/compete secondary, 2—primary/secondary special, 3—higher/postgraduate), and work categories (1—retired, 2—retired + work, 3—work) were specified as independent variables (predictors). To identify the final set of predictors, a procedure of stepwise inclusion of predictors in the model was used; the variance inflation factor (VIF) was used to assess multicollinearity, and when the critical value (VIF > 5) was exceeded, the predictor was excluded from the model. *B*—non-standardized regression coefficient; *β*—standardized regression coefficient; *p*—significance of regression coefficient; *R*^2^—total variance accounted for predictors at their stepwise inclusion in the model; Δ*R*^2^—portion of the variance accounted for by separate predictors in the model.

**Table 3:** Results of logistic regression analyses.

#	Dependent Variables	Predictors	*B*	OR	95% CI		^&^ *p*	Omnibus Test		Hosmer-Lemeshov Test	
								*χ* ^2^	*p*	*χ* ^2^	*p*
1	ZSDSIc	Sex	0.930	2.533	1.049	6.118	0.039	32.27	0.000	3.84	0.871
		FMT_day_	−0.488	0.614	0.436	0.864	0.005				
2	ZSDSIc	Sex	0.884	2.421	1.001	5.857	0.050	29.64	0.000	13.12	0.108
		FMT_dinner_	−0.400	0.671	0.476	0.945	0.023				
3	RCS Logic	FMT_day_	0.726	2.066	1.131	2.204	0.013	8.10	0.004	0.01	0.945
4	RCS Logic	FMT_dinner_	0.635	1.887	1.183	2.138	0.033	9.79	0.002	0.44	0.510

A series of binary logistic regression analyses were performed, in which sleep quality categories (codes: 0—PSQI ≤ 5 or good, 1—PSQI > 5 or bad), social jetlag categories (codes: 0—SJL < 1 h, 1—SJL ≥ 1 h), depression categories (ZSDSIc) (codes: 0—no to mild, 1—moderate to extreme), and results of logic thinking test (RCS Logic) (codes: 0—incorrect response, 1—correct response) were specified as dependent variables, while melatonin-containing food consumption per day (FMT_day_) in tertiles (codes: 1—low, 2—middle, 3—high; see details in Supplementary Information Table S1), melatonin-containing food consumption per dinner (FMT_dinner_) in tertiles (codes: 1—low, 2—middle, 3—high; see details in Supplementary Information Table S1), age, sex (codes: 1—males, 2—females), males—reference group, weight categories (BMIc) (codes: 1—underweight, 2—normal weight, 3—overweight, 4—obese), season (codes: 1—spring, 2—autumn), education categories (codes: 1—lower/compete secondary, 2—primary/secondary special, 3—higher/postgraduate), and work categories (1—retired, 2—retired + work, 3—work) were specified as independent variables (predictors). Code “0” is used in the models as a “group of comparison” for dependent variables; only significant predictors were included in the final model using the procedure “stepwise inclusion”. *B*—regression coefficient; OR—odds ratio; CI—confidence interval; ^&^ *p*—Bonferroni-corrected significance of the regression coefficient. Models’ goodness of fit was tested using Omnibus and Hosmer-Lemeshow tests.

## Data Availability

The original contributions presented in the study are included in the article/Supplementary Materials, further inquiries can be directed to the corresponding author.

## Supplementary Materials

The following supporting information can be downloaded at https://www.mdpi.com/article/10.3390/nu16071064/s1, Table S1: Descriptive statistics of FMT_day_ and FMT_dinner_.

## Abbreviations

CS: circadian system; FMT: melatonin-containing food; FMT_day_: FMT consumption for day; FMT_dinner_: FMT consumption at dinner; SWLS: Satisfaction with Life Scale; PANAS: the Positive and Negative Affect Schedule; ZSDS: the Zung Self-Rating Depression Scale; RCS: the Rapid Cognitive Screen test; MCTQ: the Munich Chronotype Questionnaire; SJL: social jetlag; PSQI: the Pittsburgh Sleep Quality Index; FFQ: Food Frequency Questionnaire; BMI: body mass index, WHtR: waist-to-height ratio.
