# Supplementary material for: The Positive Association between Melatonin-Containing Food Consumption and Older Adult Life Satisfaction, Psychoemotional State, and Cognitive Function
Source: Nutrients. 2024 Apr 5;16(7):1064. doi: 10.3390/nu16071064 (PMC11013436; doi:10.3390/nu16071064)
Supplement: Supplementary file 1 [file nutrients-16-01064-s001.zip › nutrients-2933735-supplementary.pdf]

Supplementary Materials

Table S1. Descriptive statistics of FMT_{day} and FMT_{dinner}.

**Variables**	**Tertiles**	***M***	***SD***	***Min***	***Max***
FMT_{day}	1 (Low)	360.37	189.07	30.41	691.45
	2 (Middle)	1097.70	287.45	696.22	1699.48
	3 (High)	4269.33	3259.45	1708.87	23058.22
FMT_{dinner}	1 (Low)	50.26	29.26	1.32	103.62
	2 (Middle)	237.90	88.58	106.08	427.29
	3 (High)	1741.15	2295.27	428.72	20184.06
